# B1 cells protect against *Schistosoma japonicum*–induced liver inflammation and fibrosis by controlling monocyte infiltration

**DOI:** 10.1371/journal.pntd.0007474

**Published:** 2019-06-13

**Authors:** Liang Yong, Yuanyuan Tang, Cuiping Ren, Miao Liu, Jijia Shen, Xin Hou

**Affiliations:** 1 Anhui Provincial Laboratory of Microbiology and Parasitology, Anhui Medical University, Hefei, China; 2 School of Basic Medical Sciences, Anhui Medical University, Hefei, China; London School of Hygiene and Tropical Medicine, UNITED KINGDOM

## Abstract

During *Schistosoma* infection, lack of B cells results in more severe granulomas, inflammation, and fibrosis in the liver, but the mechanisms underlying this pathology remain unclear. This study was to clarify the mechanisms underpinning the immunomodulation of B cells in mice infected with *Schistosoma japonicum* (*S*. *japonicum*). We found that B cell deficiency led to aggravated liver pathology, as demonstrated by increases in the size of the egg-associated granulomas, alanine transaminase levels, and collagen deposition. Compared with infected wild-type (WT) mice, infected B cell-deficient (μMT) mice showed increased infiltration of Ly6C^hi^ monocytes and higher levels of proinflammatory cytokines and chemokines. Furthermore, B1 cells were increased significantly in the liver of WT mice following *S*. *japonicum* infection. Adoptively transferring B1 cells, but not B2 cells, to μMT mice significantly reduced liver pathology and liver infiltration of Ly6C^hi^ monocytes. Additionally, secretion of IL-10 from hepatic B cells increased significantly in infected WT mice and this IL-10 was mainly derived from B1 cells. Adoptively transferring B1 cells purified from WT mice, but not from IL-10-deficient mice, to μMT mice significantly reduced liver pathology and liver infiltration of Ly6C^hi^ monocytes. These reductions were accompanied by decreases in the expression levels of chemokines and inflammatory cytokines. Taken together, these data indicated that after *S*. *japonicum* infection, an increased number of hepatic B1 cells secrete IL-10, which inhibits the expression of chemokines and cytokines and suppresses the infiltration of Ly6C^hi^ monocytes into the liver thereby alleviating liver early inflammation and late fibrosis.

## Author summary

Infection with *Schistosoma* results in strong granulomatous inflammation caused by parasite eggs deposited in the liver. Granuloma is defined as a significant number of immune cell infiltration around the eggs intermixed with hepatocytes, which can protect the host against liver damage. But excessive infiltration and inflammation lead to severe liver injury and fibrosis. Here we found that B1 cells accumulated in the liver after infection and released IL-10 to regulate inflammation. B1 cell-derived IL-10 inhibited the expression of chemokines and then restrained excessive infiltration of Ly6C^hi^ monocytes into the liver thereby alleviating early inflammation and later fibrosis in the liver. Our study provides insight into the immunomodulation of B1 cells in schistosomiasis and an important step towards the development of therapeutic strategies for *Schistosoma*-induced liver diseases.

## Introduction

Schistosomiasis is a chronic disease with the characteristic pathological manifestation of granulomatous lesions around parasitic eggs deposited in the liver and intestine. Granulomas are driven by a type 2 immune response and are a critical component in limiting the amount of tissue damage and preventing acute mortality [1]. However, granulomas may ultimately lead to liver fibrosis and sometimes to death in chronically infected hosts [2].

Macrophages are a major cellular component of granulomas. Both monocyte-derived and resident macrophages engage surrounding parasite eggs during infection [3, 4]. The recruitment of Ly6C^hi^ monocytes is the dominant mechanism for expanding macrophage populations in the *Schistosoma*-infected liver [3]. Recruitment of Ly6C^hi^ monocytes to inflammatory sites depends on the interactions between chemokine (C-C motif) ligands (CCLs) and their receptors (CCRs), including CCL2-CCR2, CCL1-CCR8, CCL3/4/5-CCR1/5, and CXCL10-CXCR3 interactions [5–8]. The CCL2-CCR2 axis is a key manner to recruit Ly6C^hi^ monocytes into the liver, and major producers of CCL2 in the liver are hepatic stellate cells and Kupffer cells [8–10]. The number of Ly6C^hi^ monocytes in the livers of *Schistosoma*-infected CCR2-deficient mice is significantly reduced compared with that in infected WT mice [3]. Liver recruitment of Ly6C^hi^ monocytes has been documented in viral infection, sterile heat injury to the liver, and ischemia-reperfusion damage when Ly6C^hi^ monocyte-derived macrophages have an M1 or proinflammatory phenotype and aggravate liver injury and fibrosis by releasing proinflammatory and profibrotic factors [9, 10]. In schistosomiasis, Ly6C^hi^ monocytes in granulomas respond to Th2-cell derived IL-4 and IL-13 to exhibit an arginase 1-positive, resistin-like molecule alpha-positive and chitinase-like 3-positive M2 phenotype or an alternatively activated macrophage (AAM) phenotype [11]. Studies using animal models have indicated that AAMs are essential to prevent fatal intestinal damage and sepsis during acute schistosomiasis; however, AAMs can also produce a variety of factors to recruit and activate fibroblasts, which contribute to the development of fibrosis [2, 12]. Depletion of macrophages/monocytes attenuates liver and lung granuloma formation and tissue fibrosis after *Schistosoma* infection [3, 13]. Thus, preventing excessive monocyte infiltration is important for tissue repair and host survival in chronic schistosomiasis. Nevertheless, despite the clear and well-documented roles of monocytes and macrophages in schistosomiasis, little is known about the mechanisms underlying regulation of monocyte infiltration.

Infection with *Schistosoma* induces IL-10-producing B cells, a relatively new member in the network of regulatory immune cells [14, 15]. *Schistosoma mansoni* (*S*. *mansoni*)-infected B cell-deficient μMT mice show more extensive hepatic granulomas and fibrosis than WT mice [16–18], but the mechanisms underpinning this difference are unclear. In mice, two major populations of B cells exist: B1 cells and B2 cells. On the basis of CD5 expression, B1 cells can be further subdivided into B1a (CD5^+^) and B1b (CD5^−^) subsets [19–21]. The B1 cells reside mainly in the peritoneal and pleural cavities, with low frequency (<5%) in the spleen. The B1a cells spontaneously secrete natural IgM antibodies, which bind self-antigens, bacterial cell wall components, or viruses [22, 23]. The B1a cells also spontaneously secrete IL-10, which regulates acute and chronic inflammatory diseases [19]. In the present study, we investigated the cross talk between B1 cells and monocytes to understand their roles in the pathogenesis of schistosomiasis. By using a murine model of *S*. *japonicum* infection, we demonstrated that B1 cells suppress granulomatous inflammation and liver fibrosis by regulating Ly6C^hi^ monocyte infiltration. We also found that IL-10 was required for B1 cells to downregulate the expression of chemokines and cytokines that attract monocytes. Understanding this immunomodulatory role of B1 cells in schistosomiasis may lead to the development of therapeutic strategies for *Schistosoma*-induced liver disease.

## Results

### *S*. *japonicum*-induced liver pathology is more severe in B cell-deficient (μMT) mice

**Fig 1:**
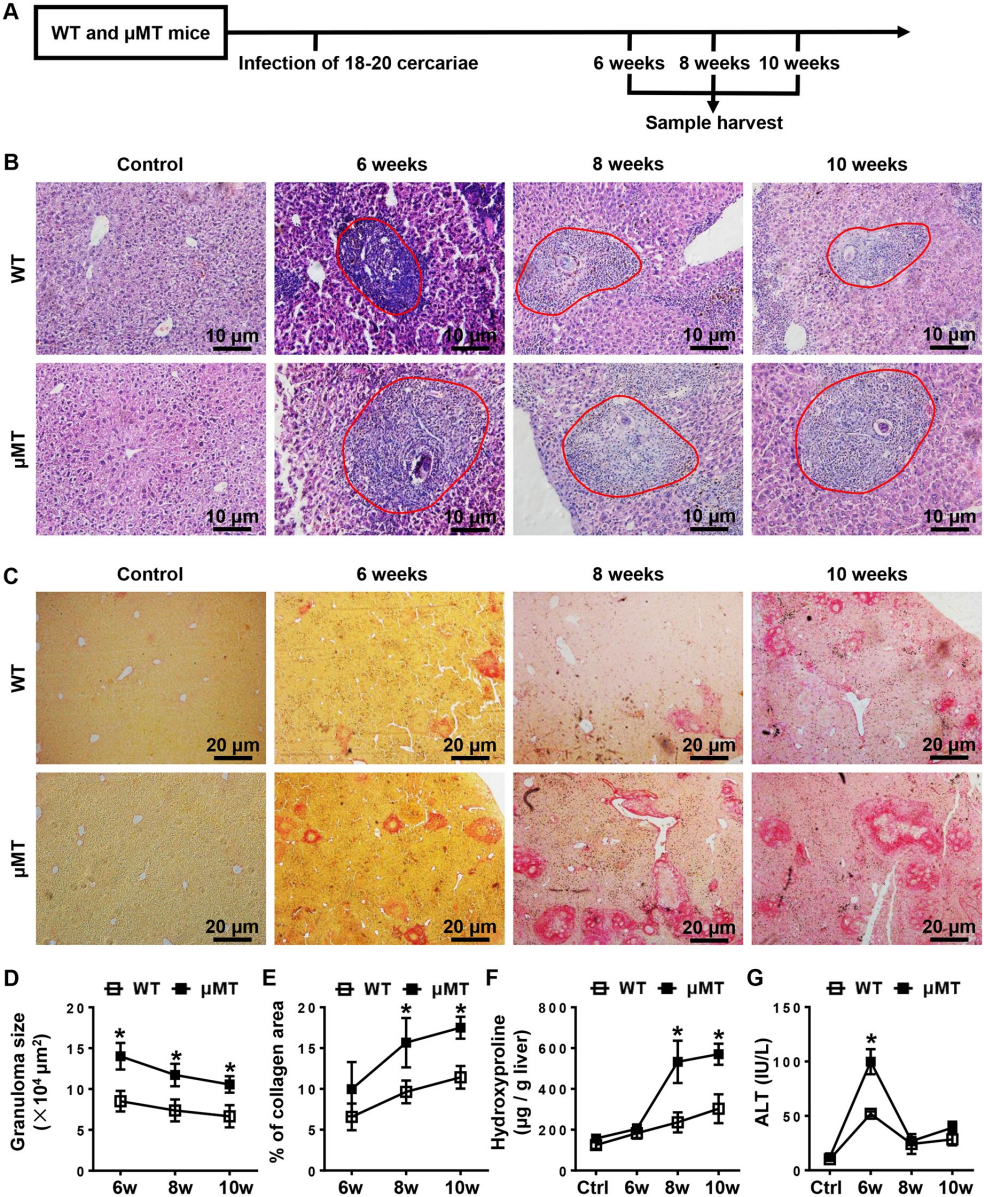
Mice lacking B cells exhibit more severe liver pathology than WT mice after *S*. *japonicum* infection. **(A)** Schematic representation of the model of *S*. *japonicum* infection. WT mice and μMT mice were infected with 18–20 cercariae of *S*. *japonicum*, and liver samples from these mice were harvested at the times indicated after infection. Comparisons were made with uninfected control mice. **(B, C)** Representative graphs of H&E staining **(B)** and picrosirius red staining **(C)** of liver specimens. **(B)** All images were taken at 200 × magnification. The red outlined areas indicate granulomas. **(C)** All images were taken at 40 × magnification. **(D-G)** Statistical analysis of granuloma sizes **(D)**, proportion of collagen areas **(E)**, amount of hepatic hydroxyproline **(F)**, and levels of serum ALT **(G)**. Data represent mean ± SD; n = 8–10 per time point from two independent experiments. *p < 0.05, versus WT mice.

To assess the role of B cells in the liver pathology associated with schistosomiasis, we infected μMT mice and WT mice with *S*. *japonicum* and harvested samples at the indicated times (Fig 1A). We found that the sizes of the hepatic granulomas after infection in μMT mice were greater than those in WT mice (Fig 1B and 1D). Liver fibrosis was measured using picrosirius red staining and hydroxyproline levels. The results showed that both the proportion of the collagen area and the hepatic hydroxyproline levels in μMT mice 8 weeks and 10 weeks after infection were increased compared with those in WT mice (Fig 1C, 1E and 1F), indicating that μMT mice exhibited increased hepatic fibrosis. In addition, serum ALT levels were significantly higher in μMT mice 6 weeks after infection (Fig 1G), suggesting that liver injury is more severe in μMT mice than in WT mice. The more severe liver pathology in μMT mice was not due to an increased burden of infection because the numbers of eggs observed in the liver samples did not differ significantly between μMT mice and WT mice (S1 Fig). Together, these data reveal an important role for B cells in attenuating *S*. *japonicum* egg-induced granuloma formation, hepatic injury, and hepatic fibrosis.

### Macrophages were markedly increased in the liver of μMT mice after *S*. *japonicum* infection

**Fig 2:**
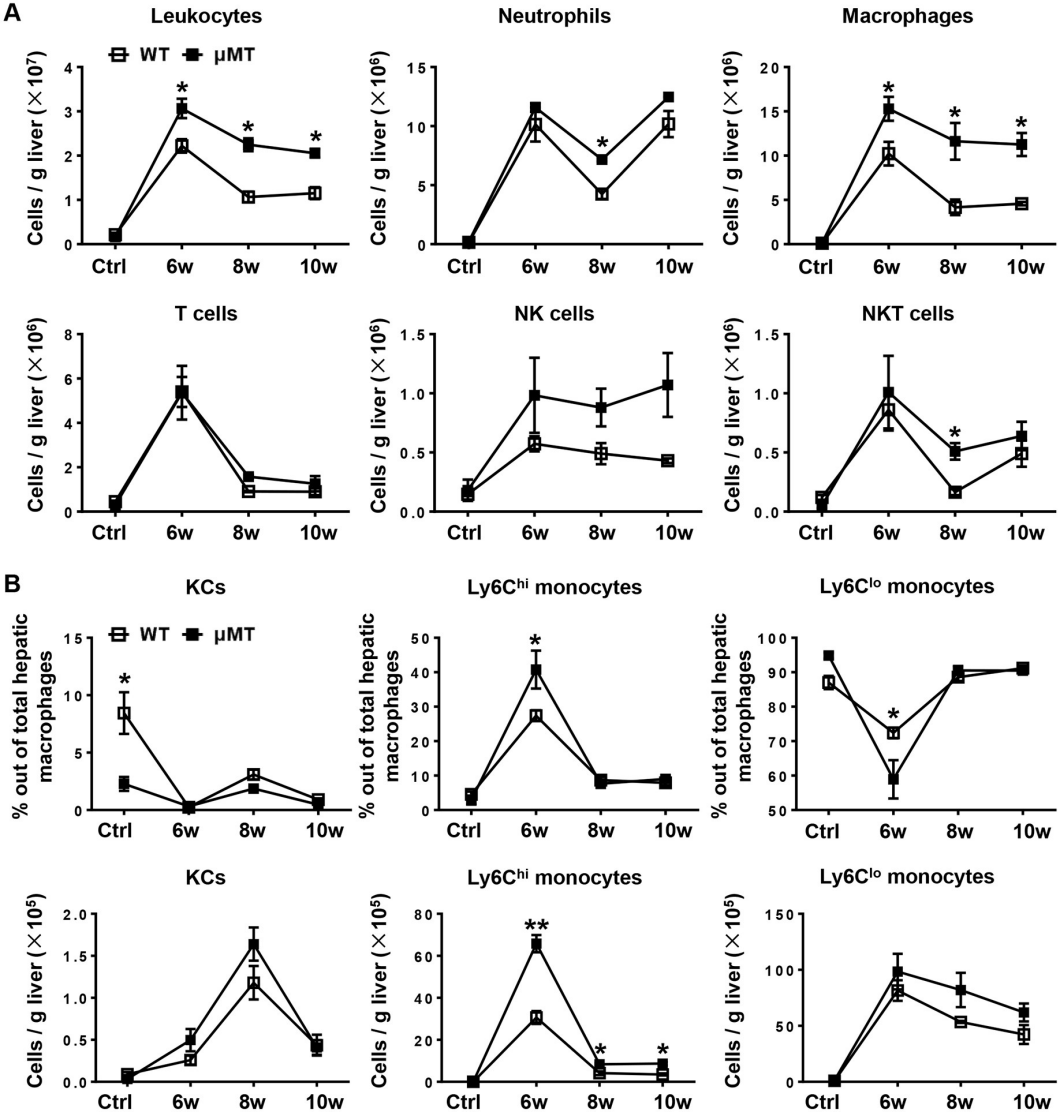
B cell deficiency results in increased number of macrophages in the liver. **(A)** The infiltration of hepatic leukocytes (CD45^+^), macrophages (CD45^+^CD11b^+^F4/80^+^), neutrophils (CD45^+^CD11b^+^Ly6G^+^), T cells (CD45^+^CD3^+^NK1.1^−^), NK cells (CD45^+^CD3^−^NK1.1^+^), and NKT cells (CD45^+^CD3^+^NK1.1^+^) after infection were quantified by flow cytometric analysis. Controls (Ctrl) were uninfected mice. **(B)** Graphical summary showing percentages of KCs (CD11b^lo^F4/80^hi^), Ly6C^hi^ monocytes (CD11b^hi^F4/80^lo^ly6C^hi^), and Ly6C^lo^ monocytes (CD11b^hi^F4/80^lo^ly6C^lo^) out of total hepatic macrophages (CD45^+^CD11b^+^F4/80^+^). **(C)** Absolute numbers of KCs, Ly6C^hi^ monocytes, and Ly6C^lo^ monocytes in WT mice and μMT mice. Data represent mean ± SD; n = 8–10 mice per time point from two independent experiments. *p < 0.05, **p < 0.01, versus WT mice.

**Fig 3:**
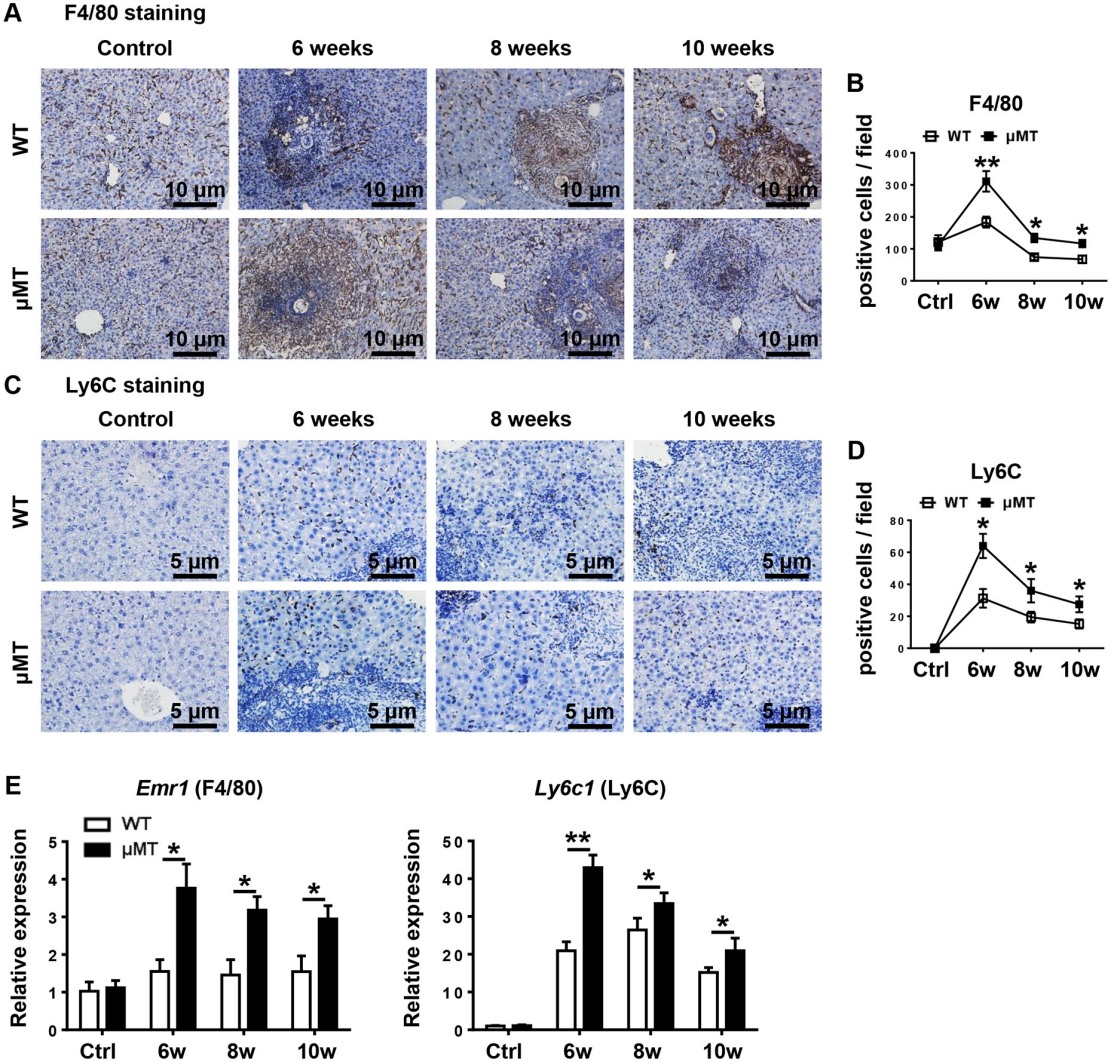
F4/80 and Ly6C expression are increased in the liver of μMT mice after *S*. *japonicum* infection. WT mice and μMT mice were infected with 18–20 cercariae of *S*. *japonicum*, and liver samples from these mice were harvested at the times indicated after infection. Comparisons were made with uninfected control mice. **(A, C)** Liver sections were stained with F4/80 **(A)**, and Ly6C **(C)**. Original magnification, × 200 for F4/80, × 400 for Ly6C. **(B, D)** Numbers of F4/80- and Ly6C-positive cells per field are counted. **(E)** Relative mRNA expression of F4/80 and Ly6C in liver tissues was determined by quantitative real-time PCR. Genes were normalized to the housekeeping gene *Actb* as an internal control. Data represent mean ± SD; n = 8–10 mice per time point from two independent experiments. *p < 0.05, **p < 0.01, versus WT mice.

To analyze the cellular components in the granulomas, we isolated hepatic leukocytes and used flow cytometry to detect cell phenotypes. Representative dot plots showing flow cytometric identification of leukocyte subsets in murine liver were shown in S2 Fig. The results showed that compared with those of WT mice the numbers of leukocytes (CD45^+^) and total macrophages (CD45^+^CD11b^+^F4/80^+^) were markedly increased in μMT mice during all stages of infection. The numbers of neutrophils (CD45^+^CD11b^+^Ly6G^+^), and of NKT cells (CD45^+^CD3^+^NK1.1^+^), were modestly but significantly increased in μMT mice 8 weeks after infection, while the numbers of T cells (CD45^+^CD3^+^NK1.1^−^) and NK cells (CD45^+^CD3^−^NK1.1^+^) showed no significant changes between WT mice and μMT mice (Fig 2A). Hepatic macrophages consist of distinct populations termed resident Kupffer cells (KCs) and monocyte-derived macrophages. Infiltrating monocytes can be further identified as two distinct subsets: proinflammatory Ly6C^hi^ monocytes and restorative Ly6C^lo^ monocytes [24]. Through flow cytometric analyses, we found that a larger number of proinflammatory Ly6C^hi^ monocytes infiltrated into the liver in μMT mice than that in WT mice after infection (Fig 2B). By contrast, the numbers of KCs and Ly6C^lo^ monocytes were similar in μMT mice and WT mice (Fig 2B). To further confirm macrophages were increased in the liver of μMT mice after infection, we performed immunohistochemical staining for F4/80 and Ly6C. As illustrated in Fig 3A–3D, immunohistochemistry analyses revealed that *S*. *japonicum*-infected μMT mice had much higher number of F4/80-expressing cells and Ly6C-expressing cells in the liver compared to *S*. *japonicum*-infected WT mice. In addition, quantitative RT-PCR analyses also revealed that expression of F4/80 and Ly6C in liver tissues was higher in *S*. *japonicum*-infected μMT mice than those of WT mice (Fig 3E). Additionally, we analyzed other specific markers CLEC4F and IBA-1 for KCs and monocytes respectively using immunofluorescent staining, showing that CLEC4F^−^IBA-1^+^ monocytes were increased in the livers of μMT mice 6 weeks and 8 weeks after infection than that in WT mice (S3 Fig).

**Fig 4:**
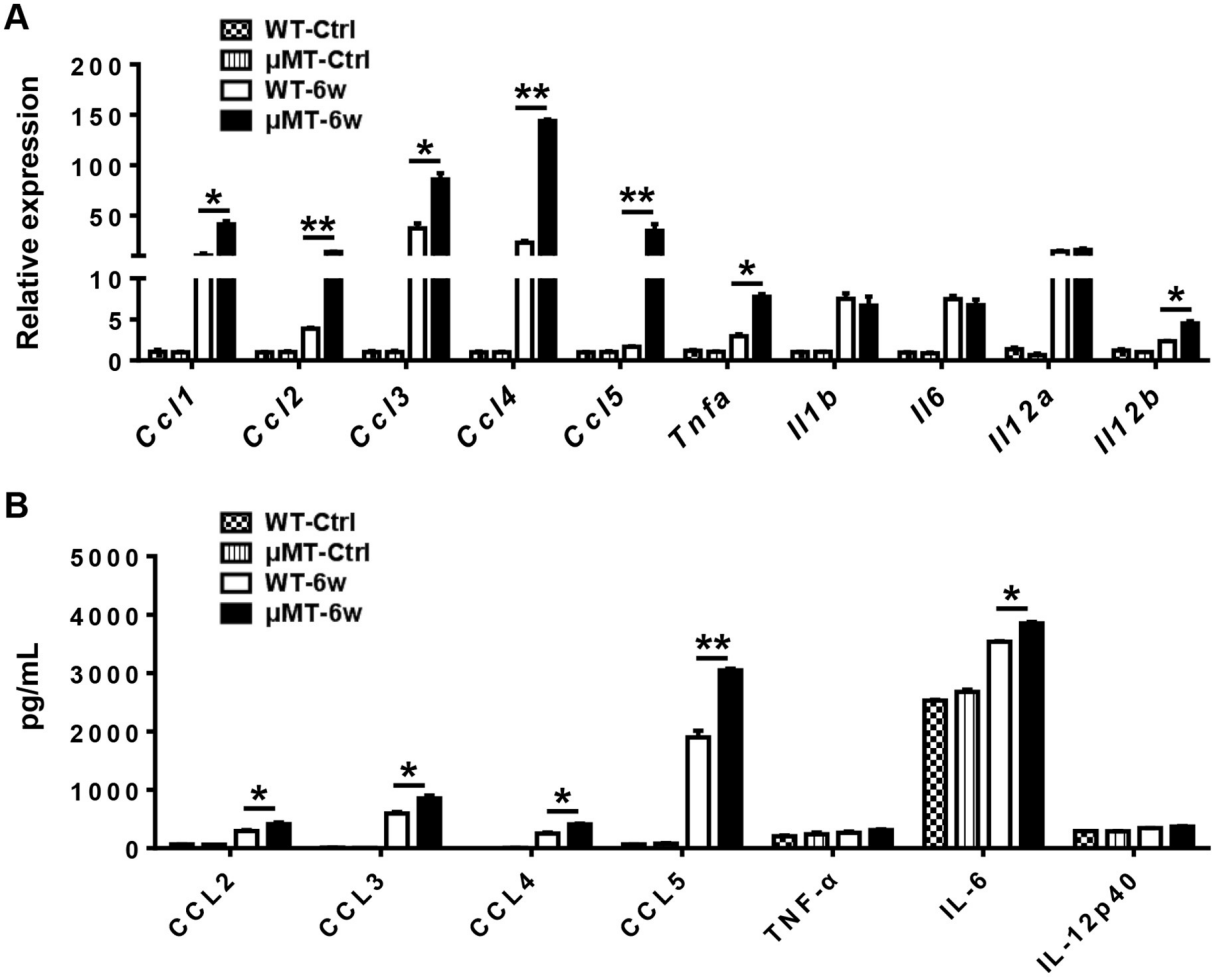
Inflammatory cytokines and chemokines are increased in the liver of μMT mice after *S*. *japonicum* infection. **(A)** Relative gene expression of chemokines (*Ccl1*, *Ccl2*, *Ccl3*, *Ccl4*, and *Ccl5*) and inflammatory cytokines (*Tnfa*, *Il1b*, *Il6*, *Il10*, *Il12a*, and *Il12b*) in the livers of WT mice and μMT mice uninfected and 6 weeks after infection. Genes were normalized to the housekeeping gene *Actb* as an internal control. **(B)** Protein levels of CCL2, CCL3, CCL4, CCL5, TNF-α, IL-6, and IL-12p40 in the livers of WT mice and μMT mice uninfected and 6 weeks after infection. Data represent mean ± SD; n = 8–10 mice per group from two independent experiments. *p < 0.05, **p < 0.01.

We hypothesized that the increased macrophage number in the liver of μMT mice after infection reflects either enhanced monocyte production or increased monocyte recruitment. Because Ly6C^hi^ monocytes are derived from the bone marrow and circulate in the blood [25], we analyzed Ly6C^hi^ monocytes in the peripheral blood. We found no significant difference in the number of circulating Ly6C^hi^ monocytes after infection, despite somewhat higher counts in uninfected μMT mice (S4 Fig), which suggests that monocyte production does not account for the increased monocyte number in the liver of μMT mice. To evaluate recruitment, we used quantitative RT-PCR assays to examine the gene expression levels of chemokines that attract monocytes in the liver samples of WT mice and μMT mice uninfected and 6 weeks after infection. The expression levels of *Ccl1*, *Ccl2*, *Ccl3*, *Ccl4*, and *Ccl5* were higher in the liver of μMT mice 6 weeks after infection than in WT mice and there is no difference in basal line levels between μMT mice and WT mice that are uninfected (Fig 4A). We also detected the protein levels of some key chemokines. The protein levels of CCL2, CCL3, CCL4, and CCL5 in the liver of μMT mice 6 weeks after infection were significantly increased compared with those of WT mice (Fig 4B). These data suggest that mobilization and recruitment, rather than production, accounted for differences in monocyte infiltration. Therefore, we speculated that B cells limit monocyte influx by suppressing the expression of chemokines during the acute stage of *S*. *japonicum* infection.

Ly6C^hi^ monocytes preferentially express inflammatory cytokines [26]. Thus, we compared the expression of selected cytokines in the livers of μMT mice and WT mice after *S*. *japonicum* infection. The gene expression levels of *Tnfa* and *Il12b* and the protein levels of IL-6 in the livers of μMT mice 6 weeks after infection were significantly higher than those in WT mice (Fig 4).

### B1 cells increase significantly in the liver and decrease in the peritoneal cavity (PC) after infection

**Fig 5:**
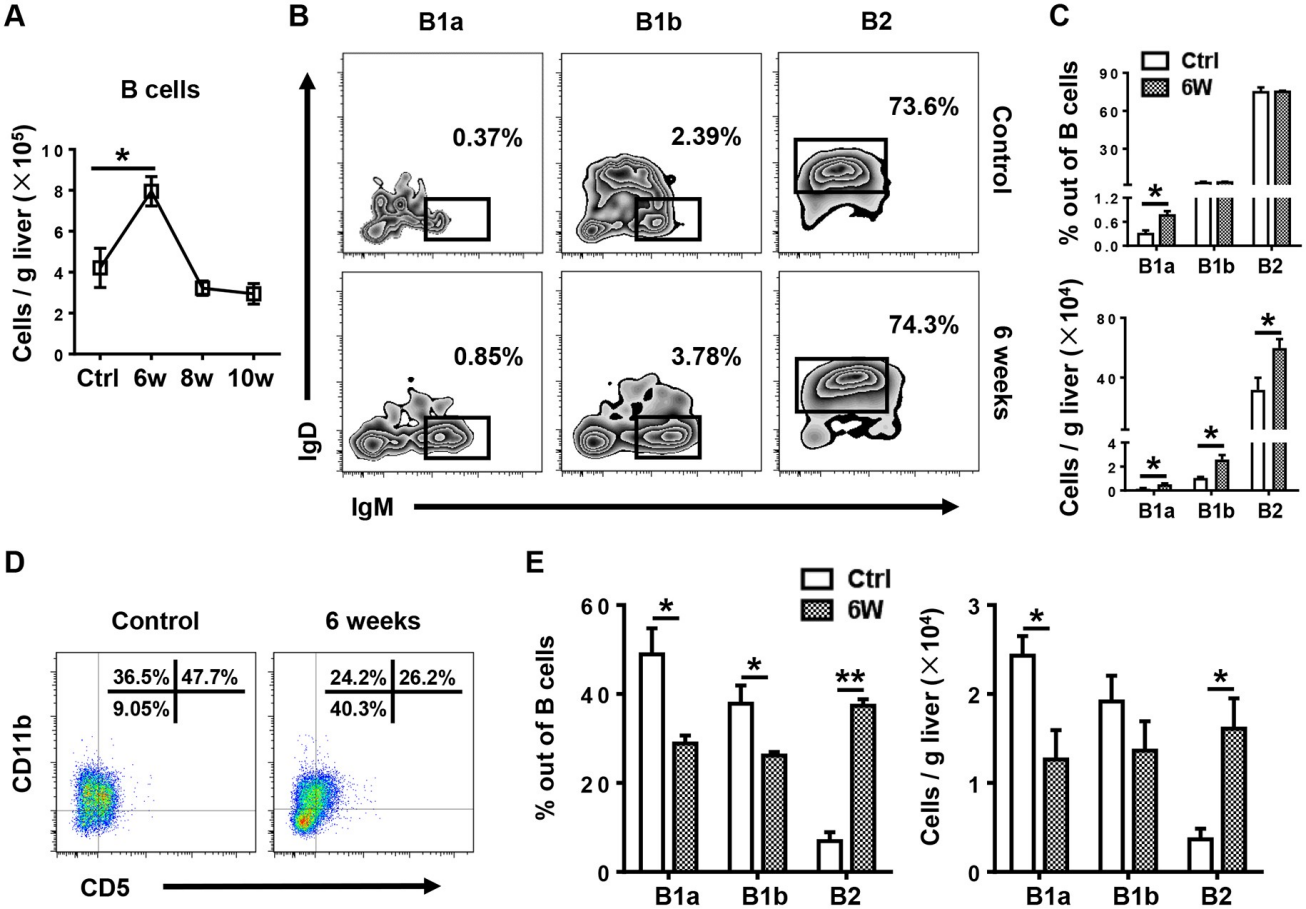
Hepatic B1 cells increase whereas PC B1 cells decrease after *S*. *japonicum* infection. **(A)** Number of B cells (CD3^−^CD19^+^) in WT mice at the times indicated after infection. **(B)** Representative flow cytometry plots of hepatic B1a (CD3^−^CD19^+^CD5^+^CD23^−^IgM^hi^IgD^lo^), B1b (CD3^−^CD19^+^CD5^−^CD23^−^IgM^hi^IgD^lo^), and B2 (CD3^−^CD19^+^CD5^−^CD23^+^IgM^lo^IgD^hi^) cells in WT mice 6 weeks after infection. **(C)** Graphical summary showing the percentages of B1a, B1b, and B2 cells out of total B cells (top panel) and the number of indicated subsets (bottom panel) in the livers of WT mice without infection (Ctrl) and 6 weeks after infection. **(D)** Representative flow cytometry plots of PC B1a (CD3^−^CD19^+^CD5^+^CD11b^+^), B1b (CD3^−^CD19^+^CD5^−^ CD11b^+^), and B2 cells (CD3^−^CD19^+^ CD5^−^CD11b^−^) in WT mice without infection and 6 weeks after infection. **(E)** The percentages of B1a, B1b, and B2 cells out of total B cells (left panel) and number of indicated subsets (right panel) in the PC of WT mice without infection and 6 weeks after infection. Data represent mean ± SD; n = 8–10 mice per group from two independent experiments. *p < 0.05, **p < 0.01.

To investigate the role and mechanism of B cell action in this murine model, we first determined the number of total B cells in the liver during infection. We found that the B cell number was significantly increased 6 weeks after infection in WT mice (Fig 5A). Further analysis of hepatic B cell subsets showed that both the percentage and number of hepatic B1a cells were markedly increased 6 weeks after infection, and the numbers of B1b cells and B2 cells were also increased (Fig 5B and 5C). In addition, we found that both the percentage and number of PC B1a cells were markedly decreased 6 weeks after infection, whereas the percentage and number of PC B2 cells were increased (Fig 5D and 5E). These data suggest that the increased B1 cells in the liver after infection might been recruited from the PC. The gating strategies for liver and PC B cell subsets were shown in S5 Fig [19]. To provide further support for this finding, we conducted adoptive cell transfer experiments. B cell-deficient μMT mice were infected with *S*. *japonicum* and then were intraperitoneally injected with uninfected WT mice-derived PC B cells or PBS 4 weeks after infection. Samples were harvested 6 weeks after infection (S6A Fig). The purity of WT mice-derived PC B cells was more than 95% (S6B Fig). After the cell transfer, B cell subsets could be detected in the PC and liver of μMT mice. Compared with those in donor WT mice, the percentage of B1a cells in the PC was lower in the recipient μMT mice, whereas the percentage of B1a cells in the liver was higher, which was consistent with our observations in the infected WT mice (S6C–S6F Fig).

### B1 cells protect against *S*.*japonicum* infection-induced liver pathology

**Fig 6:**
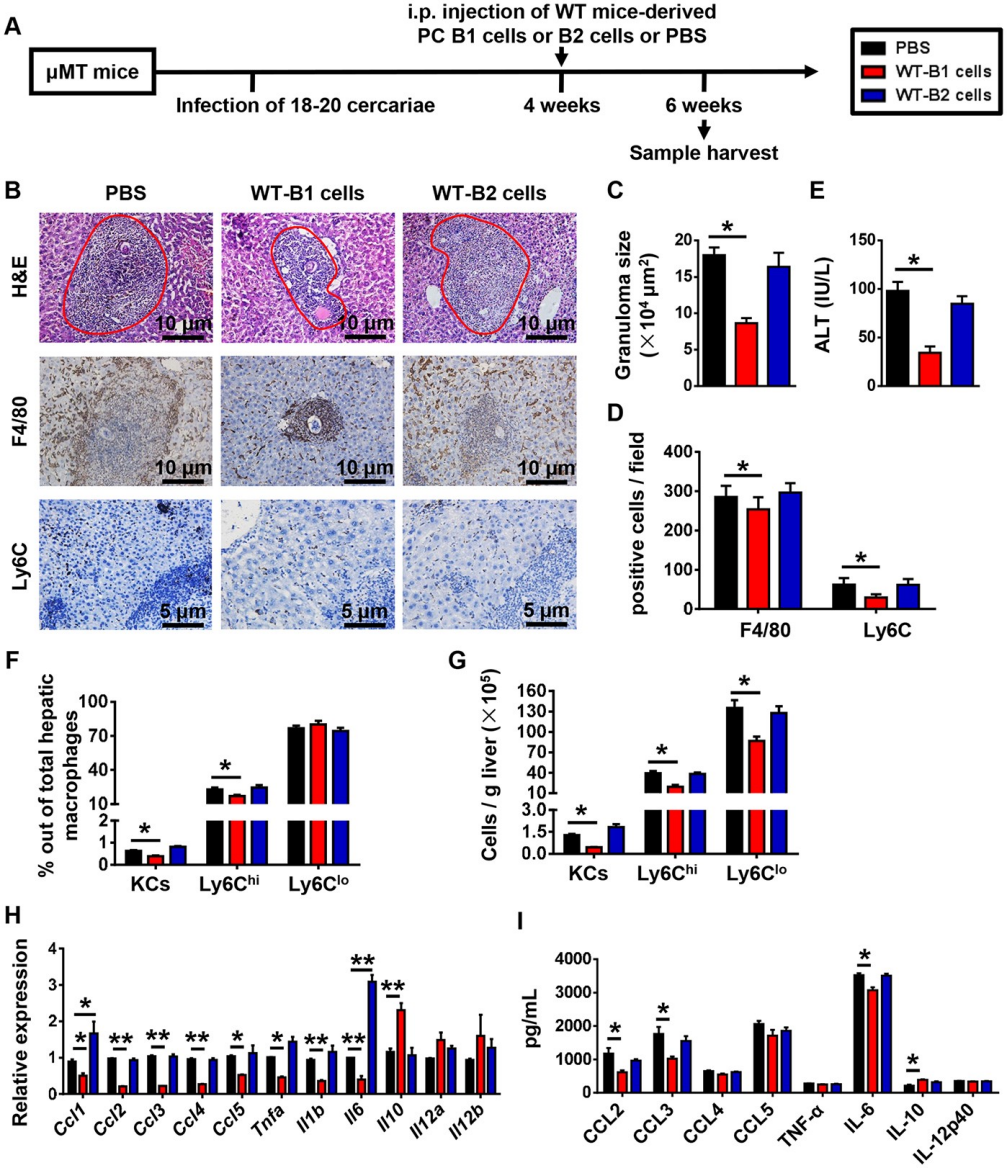
Adoptive transfer of B1 cells attenuates *S*. *japonicum*-induced liver pathology in μMT mice. **(A)** Schematic representation of the model of mice treatment. The μMT mice were infected with 18–20 cercariae of *S*. *japonicum*. FACS-sorted PC B1 cells (2 × 10^6^ cells) or B2 cells (2 × 10^6^ cells) from uninfected WT mice were adoptive transferred into μMT mice 4 weeks after infection. Mice were sacrificed 6 weeks after infection. **(B)** Representative images of H&E, F4/80, and Ly6C stained liver tissues. Original magnification, × 200 for F4/80, × 400 for Ly6C. **(C-E)** Statistical analysis of hepatic granuloma sizes **(C)**, numbers of F4/80- and Ly6C-positive cells per field **(D)**, and serum ALT levels **(E)**. **(F)** Graphical summary showing percentages of indicated cell subsets out of total hepatic macrophages in infected μMT mice after cell transfer assayed by flow cytometry. **(G)** Numbers of indicated cell subsets in the liver of infected μMT mice after cell transfer analyzed by flow cytometry. **(H)** Quantitative PCR analysis of chemokine and inflammatory cytokine gene expression levels in the liver. **(I)** Hepatic chemokine and inflammatory cytokine protein expression levels were examined by CBA and ELISA. Data represent mean ± SD; n = 5–7 per group from two independent experiments. *p < 0.05, **p < 0.01.

To examine whether B1 cells or B2 cells protect against *S*.*japonicum* infection-induced liver pathology, we adoptively transferred PC B1 cells or B2 cells from WT mice into *S*. *japonicum*-infected μMT mice (Fig 6A). The μMT mice that received B1 cells showed decreases in the granuloma sizes, ALT levels, numbers of F4/80-expessing cells, Ly6C^hi^ and Ly6C^lo^ monocytes, and the expression levels of hepatic chemokines and inflammatory cytokines compared with control mice receiving PBS (Fig 6B–6I). However, μMT mice which received B2 cells had no difference in the granuloma sizes, numbers of F4/80-expessing cells, Ly6C^hi^ and Ly6C^lo^ monocytes, and expression levels of chemokines and inflammatory cytokines in the liver compared with μMT mice which received PBS (Fig 6B–6I).

### IL-10 is indispensable for B1 cell protection against *S*. *japonicum* infection-induced liver pathology

**Fig 7:**
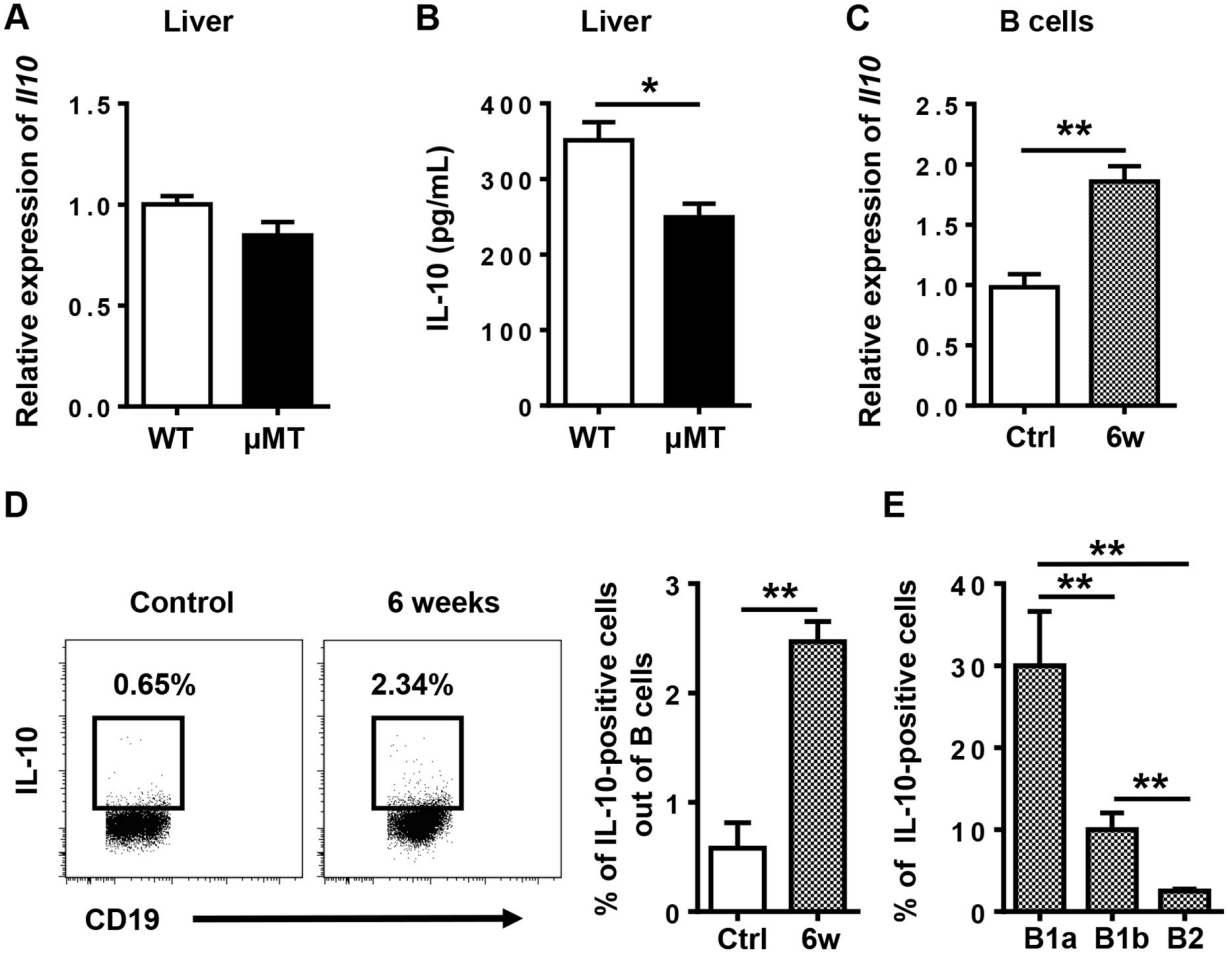
IL-10 expression is increased in the liver and in B cells after *S*. *japonicum* infection. **(A)** Relative *Il10* gene expression in the livers of WT mice and μMT mice 6 weeks after infection. **(B)** IL-10 protein levels in the livers of WT mice and μMT mice 6 weeks after infection. **(C)** Quantitative PCR analysis of *Il10* in sorted hepatic B cells. **(D)** The frequency of IL-10-positive cells out of total B cells in the livers of WT mice was examined by flow cytometry. **(E)** Graphical summary showing percentage of IL-10-positive cells out of indicated cell subsets in WT mice 6 weeks after infection. Data represent mean ± SD; n = 8–10 per group from two independent experiments. *p < 0.05, **p < 0.01.

The regulatory function of B1 cells is mediated mainly by their secretion of IL-10 [21, 27–30]. To determine whether B1 cells protect against *S*. *japonicum* infection-induced liver pathology via IL-10, we first examined IL-10 expression in the liver and B cells. The hepatic IL-10 protein levels in μMT mice were significantly lower than those of WT mice 6 weeks after infection (Fig 7A and 7B), suggesting that B cells contribute to IL-10 production in the liver after infection. Our data also showed that IL-10 expression levels in B cells were increased after infection (Fig 7C and 7D), especially in hepatic B1a cells (Fig 7E).

**Fig 8:**
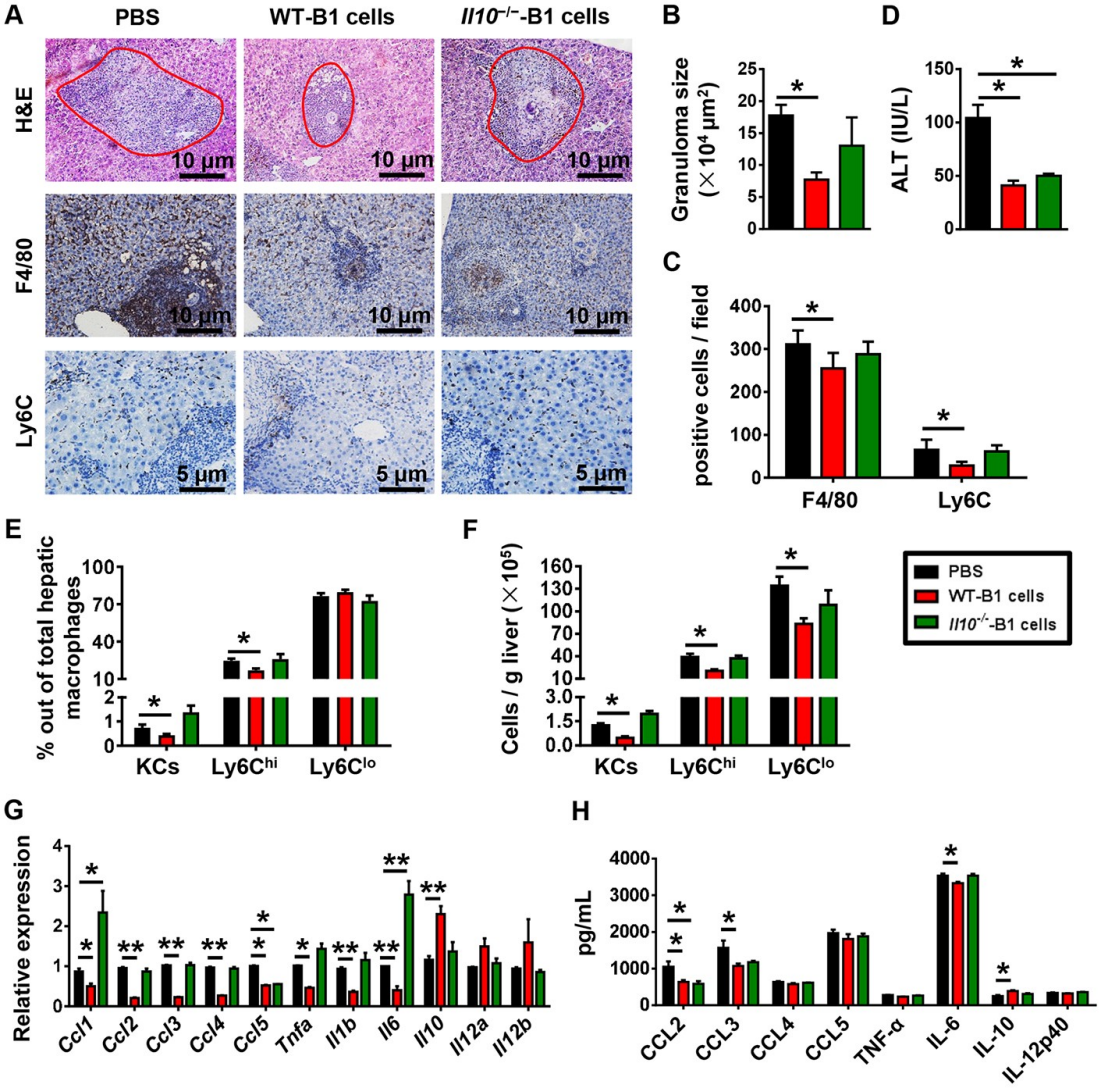
Adoptive transfer of WT PC B1 cells, but not *Il10*^−/−^ PC B1 cells, attenuates *S*. *japonicum*-induced liver pathology in μMT mice. μMT mice were infected with 18–20 cercariae of *S*. *japonicum*. The adoptive transfer of B1 cells (2 × 10^6^ cells) purified from the PC of WT or *Il10*^−/−^ mice into μMT mice was performed 4 weeks after infection. Mice were sacrificed 6 weeks after infection. **(A)** H&E staining and immunohistochemical staining for F4/80 and Ly6C of liver tissues. Original magnification, × 200 for F4/80, × 400 for Ly6C. **(B)** Statistical analysis of hepatic granuloma sizes. **(C)** Numbers of F4/80- and Ly6C-positive cells per field. **(D)** Serum ALT levels. **(E)** Graphical summary showing percentages of indicated cell subsets out of total hepatic macrophages in infected μMT mice after cell transfer assayed by flow cytometry. **(F)** Numbers of indicated cell subsets in the liver of infected μMT mice after cell transfer analyzed by flow cytometry. **(G)** Quantitative PCR analysis of chemokine and inflammatory cytokine gene expression levels in the liver. **(H)** Hepatic chemokine and inflammatory cytokine protein expression levels were examined by CBA and ELISA. Data represent mean ± SD; n = 5–7 per group from two independent experiments. *p < 0.05, **p < 0.01.

IL-10 plays a protective immunomodulatory role during schistosomiasis [16]. To assess whether IL-10 is involved in the suppressive effect of B1 cells on monocyte infiltration after *S*. *japonicum* infection, we adoptively transferred PC B1 cells from WT or *Il10*^−/−^ mice into *S*. *japonicum*-infected μMT mice. As expected, μMT mice that received WT B1 cells showed decreases in the granuloma sizes, ALT levels, numbers of F4/80-expessing cells, Ly6C^hi^ and Ly6C^lo^ monocytes, and the expression levels of hepatic chemokines and inflammatory cytokines compared with control mice receiving PBS (Fig 8A–8H). However, when μMT mice received the IL-10-deficient B1 cells, the granuloma sizes, numbers of F4/80-expessing cells, Ly6C^hi^ and Ly6C^lo^ monocytes, and expression levels of chemokines and inflammatory cytokines in the liver were not reduced compared with those in controls (Fig 8A–8H). Only the ALT levels in μMT mice receiving the transfer of IL-10-deficient B cells were decreased (Fig 8D). Collectively, these results provide evidence that the B1 regulatory subset of B cells suppress monocyte recruitment by producing IL-10 to thereby attenuate *S*. *japonicum* egg-induced liver pathology.

## Discussion

The roles of B cells in liver fibrosis remain obscure. In carbon tetrachloride (CCl_4_)-induced liver fibrosis, B cells are required for the fibrotic processes. In the CCl_4_ model, B cells serve to amplify liver fibrosis through the production of proinflammatory cytokines and chemokines [31, 32]. However, the observed mechanisms in the CCl_4_ model are not applicable to infectious liver fibrosis. The B cell-deficient mouse displays an increased hepatic fibrosis after *S*. *mansoni* infection, suggesting that B cells serve a protective role in infection-induced liver fibrosis. It has been reported that B cell could downregulate granulomatous pathology in Schistosomiasis through Fc receptor signaling. However, the mechanisms underlying B cell suppression of *Schistosoma*-induced liver pathology are still not clear.

In the present study using an *S*. *japonicum*-infected murine model, we found that B1 cells protected against *S*. *japonicum* infection-induced liver pathology by controlling liver infiltration of monocytes. In agreement with the reports using the *S*. *mansoni* infection model [16, 17], we observed markedly exacerbated hepatic granuloma formation, liver injury, and fibrosis in *S*. *japonicum*-infected B cell-deficient (μMT) mice 6 weeks post-infection. However, Fang Ji et.al found that B cell-deficient mice (OBF-1-null mice) had fewer hepatic granuloma numbers at five weeks of *S*. *japonicum* infection. At 8 weeks after *S*. *japonicum* infection, the number of granuloma in the livers of OBF-1-null mice was equal to that of WT controls [33]. We found that the number of granuloma in the livers of μMT mice were equal to that in WT mice from 6 weeks after *S*. *japonicum* infection, which was consistent with what I. Ferru et.al found in μMT mice infected with *S*. *mansoni* [18]. And the size of single-egg granuloma areas in μMT mice was bigger than that in WT mice (Fig 1). It seems that B cells are required for granuloma formation in the very early stage of *S*. *japonicum* infection, however, B cells also control granuloma sizes to avoid exceeded responses, and these different functions may be conducted by distinct B cell subsets. The accurate time of B cell function transition from promotion to suppression may vary in different B cell-deficient mice.

The B1 cells trafficked from the peritoneal cavity to the liver following infection induction. The increased B1 cells in the liver suppressed the production of chemokines, which attract monocytes, and thus controlled the recruitment of monocytes. The B1 cells played their regulatory roles via producing IL-10.

Infiltrating Ly6C^hi^ monocytes may act as a double-edged sword in liver damage. These cells express a substantial number of inflammatory cytokines and chemokines and promote liver inflammation, injury, and fibrosis in the initiation and progression of various types of liver injury, including acute viral infection, hepatotoxicity following CCl_4_ treatment, or ischemia-reperfusion damage [24, 34, 35]. We hypothesized that recruited Ly6C^hi^ monocytes also contribute to the initial liver damage and development of fibrosis after *S*. *japonicum* infection. As expected, *S*. *japonicum*-infected μMT mice with increased Ly6C^hi^ monocyte infiltration had higher levels of ALT and liver fibrosis than WT mice (Figs 1–3). When liver injury ceases, inflammatory Ly6C^hi^ monocytes mature into Ly6C^lo^ restorative macrophages, which display increased expression of anti-inflammatory cytokines, regenerative growth factors, and matrix degrading metalloproteinase [9, 25]. During chronic *S*. *mansoni* infection, Ly6C^hi^ monocytes become AAMs in granulomas through a Ly6C^lo^ state [3, 4]. The arginase 1-expressing AAM population suppresses Th2 cytokine-driven inflammation and fibrosis in schistosomiasis [36, 37]. Thus, it is crucial to regulate monocyte recruitment and homeostasis in the liver. Our results showed that compared with WT mice, μMT mice had increased hepatic Ly6C^hi^ monocytes and chemokines attracting Ly6C^hi^ monocytes after *S*. *japonicum* infection (Figs 3 and 4), which suggests that B cells play critical roles in controlling monocyte infiltration after *S*. *japonicum* infection through negative regulation of chemokines.

*Schistosoma* infection induces IL-10-producing B cells, which are termed regulatory B cells or B10 cells [27, 30]. Currently, there are no phenotypic, transcription factors, or lineage markers that are unique to B10 cells, and B10 cells mostly overlap with B1 cells [19, 21, 28]. B1 cells can secrete IL-10 to mediate the negative regulation of inflammation, including restricting the production of proinflammatory cytokines, downregulating the expression of MHC II [38], and maintaining the suppressive function of regulatory T cells [39]. In homeostatic conditions, B1 cells are localized mainly in the PC, and they are the major population of B cells in this compartment. In response to pathogens, serosal B1a cells in body cavities migrate to neighboring lymphoid sites or tissues [19, 40]. In the present study, we found that B1 cells, especially B1a cells, migrated from the PC to the liver after *S*. *japonicum* infection, which was shown by the increased percentage and number of B1a cells in the liver and their concurrent decrease in the PC after infection (Fig 5). In addition, *S*. *japonicum*-infected μMT mice receiving the adoptive transfer of PC B cells purified from WT mice also showed a higher percentage of B1a cells in the liver and a lower percentage of B1a cells in the PC than the donor mice (S6 Fig). These data suggest that B1a cells are the major population of B cells that regulate monocyte infiltration after *S*. *japonicum* infection.

IL-10 is involved in the immunoregulatory role of B1 cells [19]. It has been reported that IL-10 plays an antifibrotic role via inhibiting the proliferation and collagen synthesis of myofibroblasts [41]. Recently, a connection between IL-10 and inflammatory chemokines has been suggested in renal fibrosis and nerve injury. After the onset of unilateral ureteral obstruction, IL-10 knockout mice show increased infiltration of inflammatory cells and cytokines, including monocyte chemoattractant protein-1, TNF-α, IL-6, IL-8, RANTES, or macrophage colony-stimulating factor, in the kidney compared with WT controls [42]. After peripheral nerve injury, IL-10 plays a role in controlling the early influx and the later efflux of macrophages out of the nerve via downregulating expression of proinflammatory chemokines and cytokines [43]. Keke C. Fairfax et.al’s finding suggested that IL10R blockade might promote protective effects within the liver through local interactions with macrophages [16]. In the present study, we observed an increased expression of IL-10 in B cells. Our flow cytometric analysis indicated that B1 cells are the major population of B cells expressing IL-10 in the *S*. *japonicum*-infected liver (Fig 7). We also found that in the absence of IL-10, the transferred PC B cells were unable to downregulate granuloma inflammation, recruitment of monocytes, or the expression of proinflammatory chemokines and cytokines in the infected μMT mice (Fig 8). These data suggest that after *S*. *japonicum* infection, B cells control the recruitment of monocytes and the expression of proinflammatory chemokines and cytokines via IL-10 production.

Regulatory T lymphocytes (Treg) also have efficient immunomodulatory functions and represent a major source of IL-10 [44]. It has been reported that schistosome infection induced a significant expansion of Treg [1]. Our results also showed that T cells were significantly increased 6 weeks after infection (Fig 3). IL-10-producing CD4^+^CD25^+^ T cells have been described regulating granuloma formation in mice [45]. We speculated that Treg may also control monocyte infiltration through IL-10.

The cross talk between B cells and monocytes observed in our study appears to be opposite to that observed in CCl_4_-induced fibrosis [31]. The difference may be that different liver microenvironments induce different B cell subsets in these two models. In the CCl_4_ model, the increased B cells in the liver produce IgG and express CD138, which may be a B2 subset. The B cells in the CCl_4_ model secrete proinflammatory cytokines and chemokines, hence, they recruit dendritic cells and Ly6C^++^ monocytes. In the present model, the B1 cells were the most substantially increased B cell subset in the *S*. *japonicum*-infected liver. These B1 cells produced IL-10, which led to the suppressed recruitment of Ly6C^hi^ monocytes.

In conclusion, our data indicated that PC B1 cells infiltrate the liver after *S*. *japonicum* infection. These B1 cells secrete IL-10, which inhibits expression of key chemokine CCL2 to limit excessive liver infiltration of Ly6C^hi^ monocytes and thereby alleviate early inflammation and later liver fibrosis.

## Materials and methods

### Ethics statement

All animal experiments were approved by the Institutional Animal Care and Use Committee at Anhui Medical University (Approval Number: LLSC20150279) and conformed to the guidelines outlined in the Guide for the Care and Use of Laboratory Animals. All infection was performed under anesthesia, and all efforts were made to minimize suffering.

### Mice and parasites

The 8- to 10-week-old male C57BL/6 wild-type (WT) mice and B cell-deficient (μMT) mice on a C57BL/6 background were purchased from the Nanjing Biomedical Research Institute of Nanjing University (Nanjing, China). The *Il10*^−/−^ mice on a C57BL/6 background were provided by Professor Zhigang Tian (University of Science and Technology of China). All mice were kept under temperature- and humidity-controlled specific-pathogen-free conditions. For infection, mice were anesthetized and percutaneously exposed to 18–20 cercariae of *S*. *japonicum* (a Chinese mainland strain) that were obtained from infected *Oncomelania hupensis* snails. At the indicated times, mice were euthanized and tissue samples were harvested for later experiments.

### Histological and immunohistochemical assay

A portion of the liver tissue was fixed in 10% formalin, embedded in paraffin, cut into 4-μm sections, and stained with H&E and picrosirius red using standard protocols. Granuloma size and collagen site were calculated using cellSens Dimension microscope software by two blinded observers. Immunohistochemical staining for F4/80 (Cell Signaling) and Ly6C (Abcam, Cambridge, MA) were performed according to the manufacturer’s instructions. The images were obtained by using OLYMPUS BX53 microscope. F4/80- and Ly6C-positive cells were counted on 20 high-power (×200) or 40 high-power (×400) fields per slide, respectively.

### Cell isolation

For isolation of peritoneal cavity (PC) cells, ice-cold PBS (5 mL) containing 2% bovine serum albumin was injected into the PC. The collected cell suspension was centrifuged and then resuspended with PBS. For isolation of hepatic leukocytes, liver samples were cut and incubated in DMEM containing 0.05% collagenase IV (Sigma), 0.002% DNase I (Sangon Biotech Co. Ltd.) and 10 mM HEPES at 37°C for 30 min. Digested liver was passed through a 74-μm nylon mesh and enzyme was inactivated by adding additional DMEM. After twice centrifugation at 50g for 2 min to remove the hepatocyte pellets, hepatic leukocytes were then isolated by 40% Percoll (GE Healthcare) at 1260g for 20 min. Liver and PC CD19^+^ B cells were sorted by PE-CD19 and anti-PE microbeads using a magnetic affinity cell sorting (MACS) system (Miltenyi Biotec). PC B1 cells and B2 cells were FACS sorted according to CD11b expression: B1 cells are CD19^+^CD11b^+^, and B2 cells are CD19^+^CD11b^−^. The purity of isolated cells was >90%.

### Adoptive transfer of PC B cells

FACS-sorted uninfected WT or *Il10*^−/−^ mice-derived PC B1 cells or B2 cells (2 × 10^6^, respectively) was intraperitoneally injected into each recipient μMT mouse 4 weeks post infection.

### Flow cytometry

The following fluorochrome-conjugated monoclonal antibodies were used in this study: anti-mouse CD3, CD5, CD11b, CD19, CD23, CD45, CD115, F4/80, Ly6C, Ly6G, NK1.1, IgM, IgD (all from BioLegend, San Diego, CA), IL-10 (BD Pharmingen). The cells were blocked with FcR blocker (BD Pharmingen) and then incubated with monoclonal antibodies to surface antigens. To detect the secretion of IL-10, cells (1 × 10^6^) were stimulated with phorbol myristate acetate (30 ng/mL) (Sigma, St. Louis, Mo.), ionomycin (1 μg/mL) (Sigma, St. Louis, Mo.), monensin (5 μg/mL) (Sigma, St. Louis, Mo.), and lipopolysaccharide (10 μg/mL) (Sigma, St. Louis, Mo.) for 4 h. Cells were incubated with monoclonal antibodies to surface antigens and then fixed and permeabilized using a Transcription Factor Staining Buffer Set (eBioscience, San Diego, CA). The cells were then incubated with antibodies to IL-10. All samples were analyzed using flow cytometry (FACSVerse system, BD Biosciences) with FlowJo (version 7.6.1) software.

### RNA isolation and quantitative PCR

Total hepatic RNA was isolated from frozen liver tissue using Trizol (Invitrogen). The MACS-sorted hepatic B cells were resuspended in Trizol, and the RNA was isolated according to the manufacturer’s instructions. First strand cDNA was synthesized from ≤500 ng of RNA using a PrimeScript RT reagent kit (TaKaRa). Quantitative PCR was performed with a StepOnePlus Real-Time PCR System (Applied Biosystems, Foster City, CA) using SYBR Premix Ex Taq II (TaKaRa). The expression levels of target genes were normalized to the housekeeping gene *Actb*. Relative expression was calculated by the 2^−ΔΔCt^ method. The primers used are given in S1 Table.

### Cytokine and chemokine assays

The protein levels of murine IL-10, CCL2, CCL3, CCL4, and CCL5 from whole liver and serum were measured using a cytometric bead assay (CBA) flex set (BD Pharmingen) according to the manufacturer’s instructions. The levels of murine IL-6, IL-12p40, and TNF-α were determined using a cytokine-specific ELISA kit (R&D Systems). Protocols were used according to the manufacturer’s instructions.

### Analyses of serum alanine transaminase levels (ALT) and hepatic hydroxyproline

For analysis of hepatic hydroxyproline, liver tissue was homogenized with an equal volume of PBS that contained a protease inhibitor cocktail (Sigma-Aldrich, St. Louis, Mo.) and centrifuged for collecting supernatants. Serum ALT and hepatic hydroxyproline levels were measured using commercially available kits (Jiancheng, Nanjing, China).

### Statistical analysis

All data are expressed as mean ± SD and were analyzed using GraphPad Prism 6.01 software. One-way ANOVA and unpaired Student’s *t* tests were used to compare variables between two groups. *P* < 0.05 was considered statistically significant.

## Supporting information

S1 Table Sequences of primers. (DOCX)

S1 Fig The numbers of eggs observed in the liver samples have no differences between WT mice and μMT mice. Partial liver tissues were digested in 10% KOH at 37°C for 3 hours, then aliquots of the suspension were counted under the microscope.
(TIF)

S2 Fig Gating strategies for identifying liver cell subsets from leukocyte populations. Representative flow cytometry plots show the gating strategy to identify hepatic neutrophils, KCs, Ly6C^hi^ monocytes, and Ly6C^lo^ monocytes **(A)**, and T cells, NK cells, and NKT cells **(B)**.
(TIF)

S3 Fig Immunofluorescent staining for CLEC4F and IBA-1. **(A)** Representative immunofluorescence microscopy images of liver sections with anti-CLEC4F (red), anti-IBA-1 (green), and DAPI (blue). **(B)** Statistics of monocyte number. Monocytes were counted on 20 high-power (×200) fields per slide.
(TIF)

S4 Fig There is no difference in the numbers of circulating Ly6C^hi^ monocytes in peripheral blood of WT mice and μMT mice. For isolation of peripheral leukocytes, blood samples were incubated with ACK Lysis Buffer (0.15 M NH_4_Cl, 10 mM KHCO_3_, 0.1 mM EDTA-2Na in H_2_O, pH 7.2–7.4) on ice for 10 min to remove red blood cells. After neutralizing and washing, the pellets were resuspended with PBS. **(A)** Gating strategy for detection of peripheral Ly6C^hi^ monocytes. **(B)** Representative flow cytometry plots of Ly6C^hi^ monocytes in peripheral blood of WT mice and μMT mice. **(C)** graphical summary showing percentage of peripheral Ly6C^hi^ monocytes out of total monocytes (left panel) and number of peripheral Ly6C^hi^ monocytes (right panel) in WT mice and μMT mice without infection (Ctrl) and 6 weeks after *S*.*japonicum* infection. Data represent mean ± SD; n = 8–10 per group from two experiments. *p < 0.05.
(TIF)

S5 Fig Gating strategies for liver and PC B cell subsets. **(A)** Representative flow cytometry plots show the gating strategy to identify hepatic B1a cells (CD3^−^CD19^+^CD5^+^CD23^−^IgM^hi^IgD^lo^), B1b cells (CD3^−^CD19^+^CD5^−^CD23^−^IgM^hi^IgD^lo^), and B2 cells (CD3^−^CD19^+^CD5^−^CD23^+^IgM^lo^IgD^hi^). **(B)** PC B1a cells were identified as CD3^−^CD19^+^CD5^+^CD11b^+^. PC B1b cells were identified as CD3^−^CD19^+^CD5^−^CD11b^+^. PC B2 cells were identified as CD3^−^CD19^+^CD5^−^CD11b^−^.
(TIF)

S6 Fig Transferred B cells migrate from PC into the liver in the recipient μMT mice. **(A)** μMT mice were infected with 18–20 cercariae of *S*. *japonicum*. MACS-sorted PC B cells (2 × 10^6^ cells) were purified from uninfected WT mice, and adoptive transferred into μMT mice 4 weeks after infection. Mice were sacrificed 6 weeks after infection. **(B)** Purity of PC B cells from WT mice after sorting. **(C, D)** Flow cytometric analysis of PC **(C)** and liver **(D)** B cell subsets after transfer in μMT mice. **(E, F)** The frequencies of B1a, B1b, and B2 cells in PC **(E)** and liver **(F)** of donor WT mice and recipient μMT mice. Data represent mean ± SD; n = 8–10 per group from two independent experiments. *p < 0.05, **p < 0.01.
(TIF)
