# Supplementary material for: B1 cells protect against Schistosoma japonicum–induced liver inflammation and fibrosis by controlling monocyte infiltration
Source: PLoS Negl Trop Dis. 2019 Jun 13;13(6):e0007474. doi: 10.1371/journal.pntd.0007474 (PMC6592576; doi:10.1371/journal.pntd.0007474)
Supplement: S1 Table — (DOCX) [file pntd.0007474.s001.docx]

**S1 Table. Sequences of primers**

| Gene | Forward primer (5′–3′) | Reverse primer (5′–3′) |
| --- | --- | --- |
| *Actb* | AGAGGGAAATCGTGCGTGAC | CAATAGTGATGACCTGGCCGT |
| *Erm1* | CATAAGCTGGGCAAGTGGTA | GGATGTACAGATGGGGGATG |
| *Ly6c1* | GCAGTGCTACGAGTGCTATGG | ACTGACGGGTCTTTAGTTTCCTT |
| *Tnfa* | ACTGGCAGAAGAGGCACTC | CTGGCACCACTAGTTGGTTG |
| *Il1b* | CTGAACTCAACTGTGAAATGC | TGATGTGCTGCTGCGAGA |
| *Il6* | ACACATGTTCTCTGGGAAATCGT | AAGTGCATCATCGTTGTTCATACA |
| *Il10* | GCTCTTACTGACTGGCATGAG | CGCAGCTCTAGGAGCATGTG |
| *Il12a* | CTGTGCCTTGGTAGCATCTATG | GCAGAGTCTCGCCATTATGATTC |
| *Il12b* | TGGTTTGCCATCGTTTTGCTG | ACAGGTGAGGTTCACTGTTTCT |
| *Ccl1* | TGCCGTGTGGATACAGGATG | GTTGAGGCGCAGCTTTCTCTA |
| *Ccl2* | CCAGCAAGATGATCCCAATG | TACGGGTCAACTTCACATTC |
| *Ccl3* | GATTCCACGCCAATTCATCG | AGGCATTCAGTTCCAGGTCA |
| *Ccl4* | TTTCTCTTACACCTCCCGGC | AGCTGCTCAGTTCAACTCCA |
| *Ccl5* | GCTGCTTTGCCTACCTCTCC | TCGAGTGACAAACACGACTGC |
