# Supplementary figures and images for: B1 cells protect against Schistosoma japonicum–induced liver inflammation and fibrosis by controlling monocyte infiltration
Source: PLoS Negl Trop Dis. 2019 Jun 13;13(6):e0007474. doi: 10.1371/journal.pntd.0007474 (PMC6592576; doi:10.1371/journal.pntd.0007474)

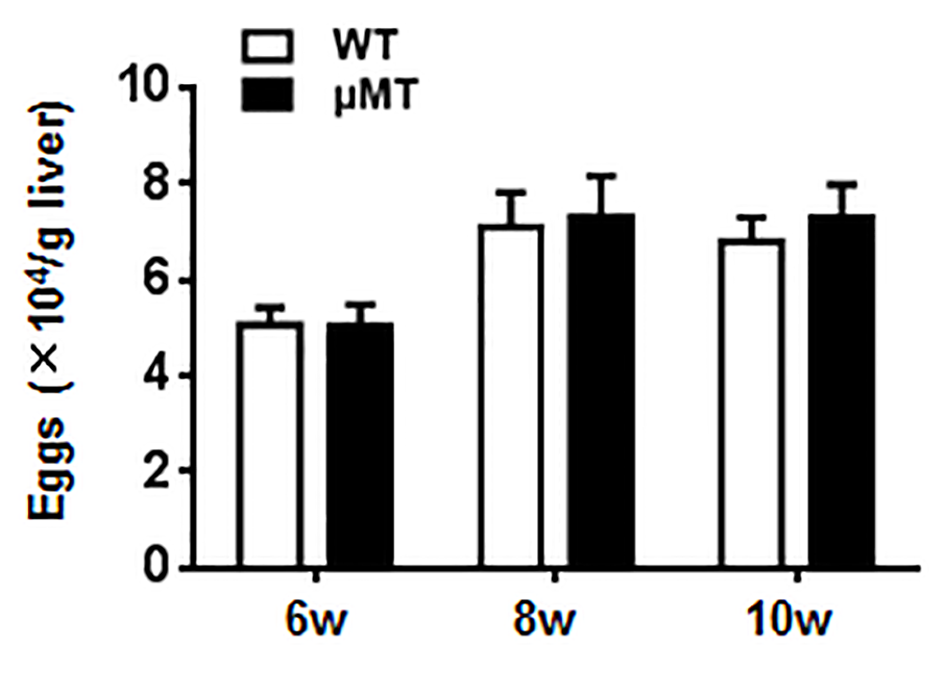

Supplement: S1 Fig — Partial liver tissues were digested in 10% KOH at 37°C for 3 hours, then aliquots of the suspension were counted under the microscope. (TIF) [file pntd.0007474.s002.tif]

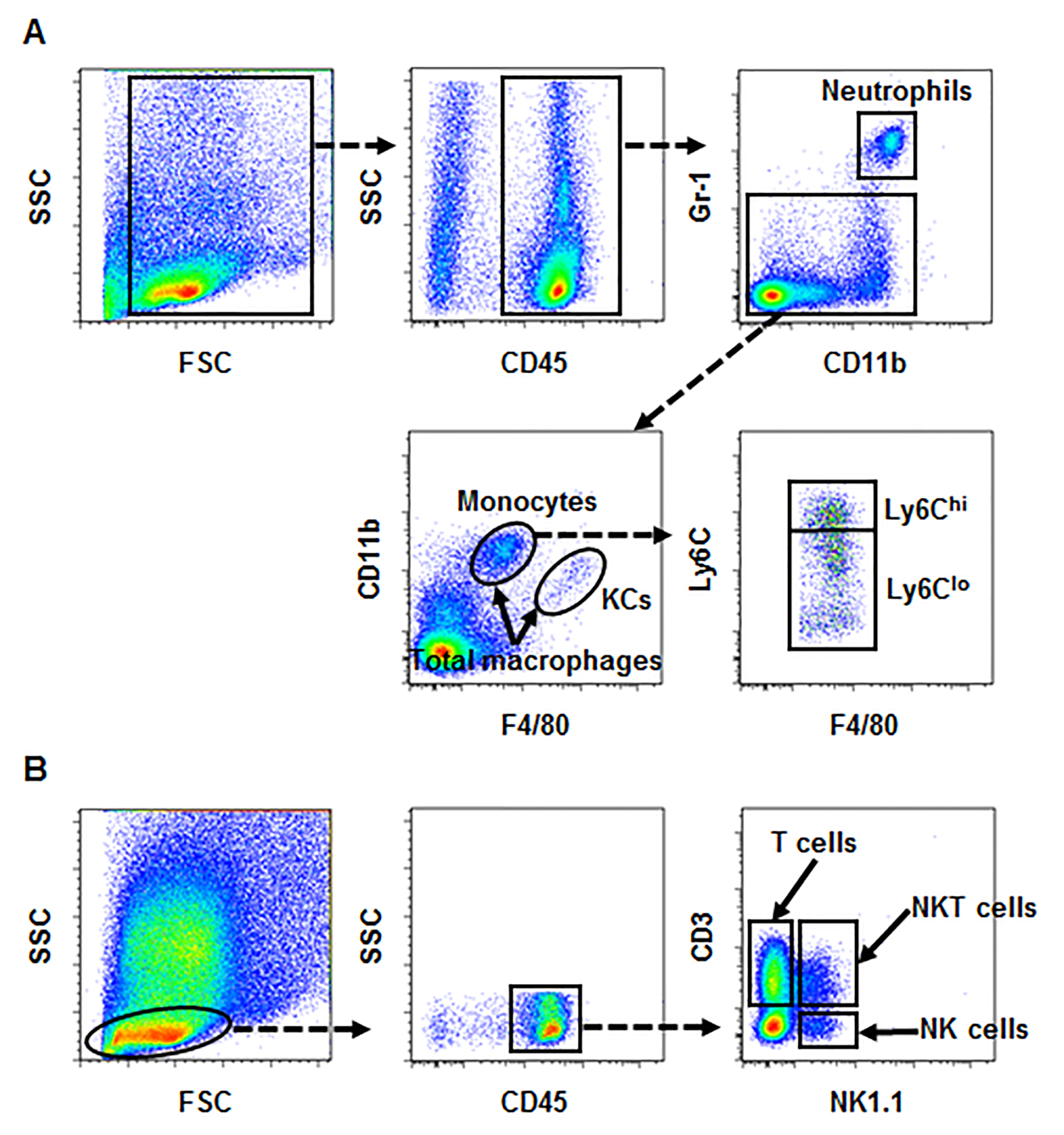

Supplement: S2 Fig — Representative flow cytometry plots show the gating strategy to identify hepatic neutrophils, KCs, Ly6Chi monocytes, and Ly6Clo monocytes (A), and T cells, NK cells, and NKT cells (B). (TIF) [file pntd.0007474.s003.tif]

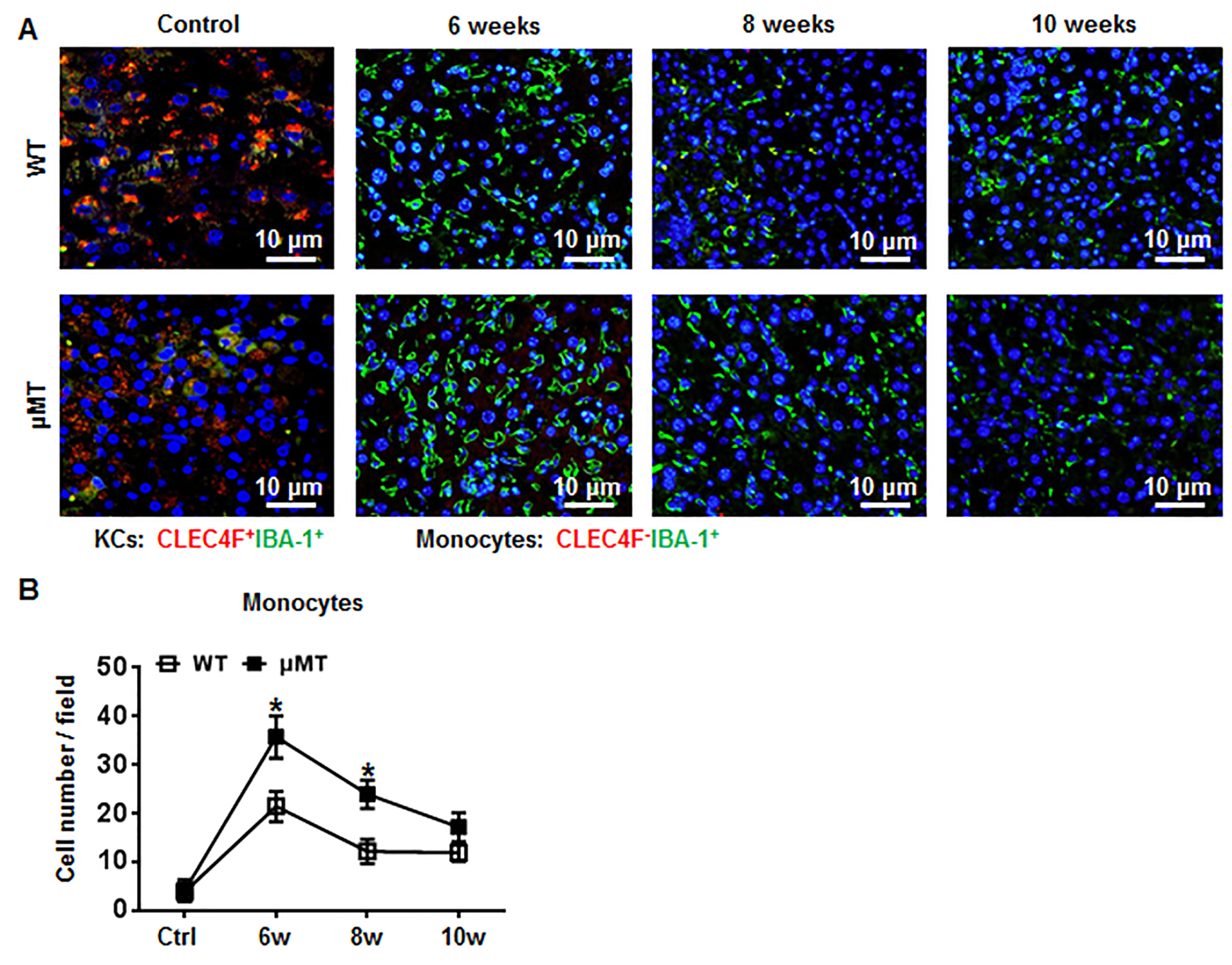

Supplement: S3 Fig — (A) Representative immunofluorescence microscopy images of liver sections with anti-CLEC4F (red), anti-IBA-1 (green), and DAPI (blue). (B) Statistics of monocyte number. Monocytes were counted on 20 high-power (×200) fields per slide. (TIF) [file pntd.0007474.s004.tif]

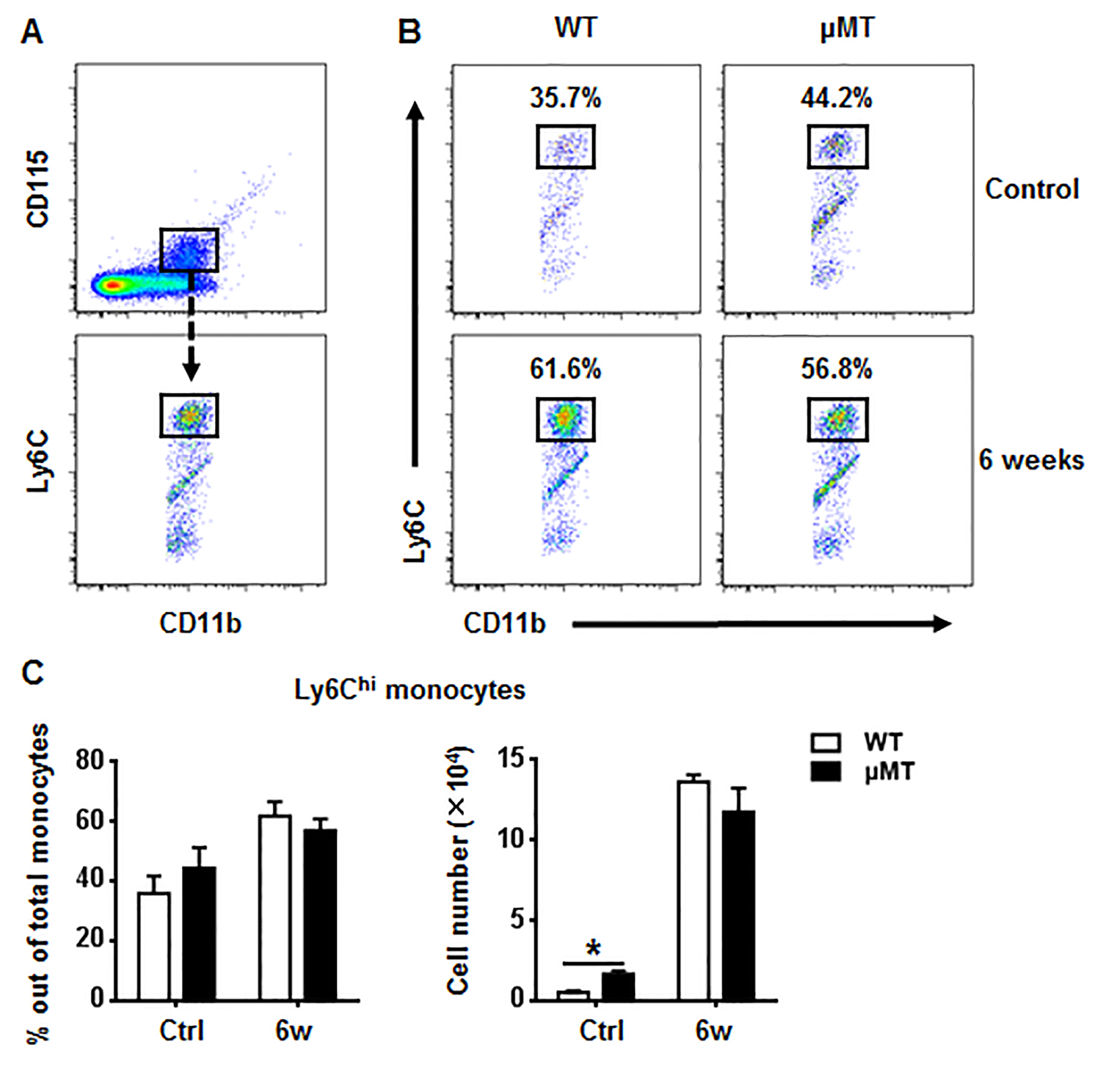

Supplement: S4 Fig — For isolation of peripheral leukocytes, blood samples were incubated with ACK Lysis Buffer (0.15 M NH4Cl, 10 mM KHCO3, 0.1 mM EDTA-2Na in H2O, pH 7.2–7.4) on ice for 10 min to remove red blood cells. After neutralizing and washing, the pellets were resuspended with PBS. (A) Gating strategy for detection of peripheral Ly6Chi monocytes. (B) Representative flow cytometry plots of Ly6Chi monocytes in peripheral blood of WT mice and μMT mice. (C) graphical summary showing percentage of peripheral Ly6Chi monocytes out of total monocytes (left panel) and number of peripheral Ly6Chi monocytes (right panel) in WT mice and μMT mice without infection (Ctrl) and 6 weeks after S.japonicum infection. Data represent mean ± SD; n = 8–10 per group from two experiments. *p < 0.05. (TIF) [file pntd.0007474.s005.tif]

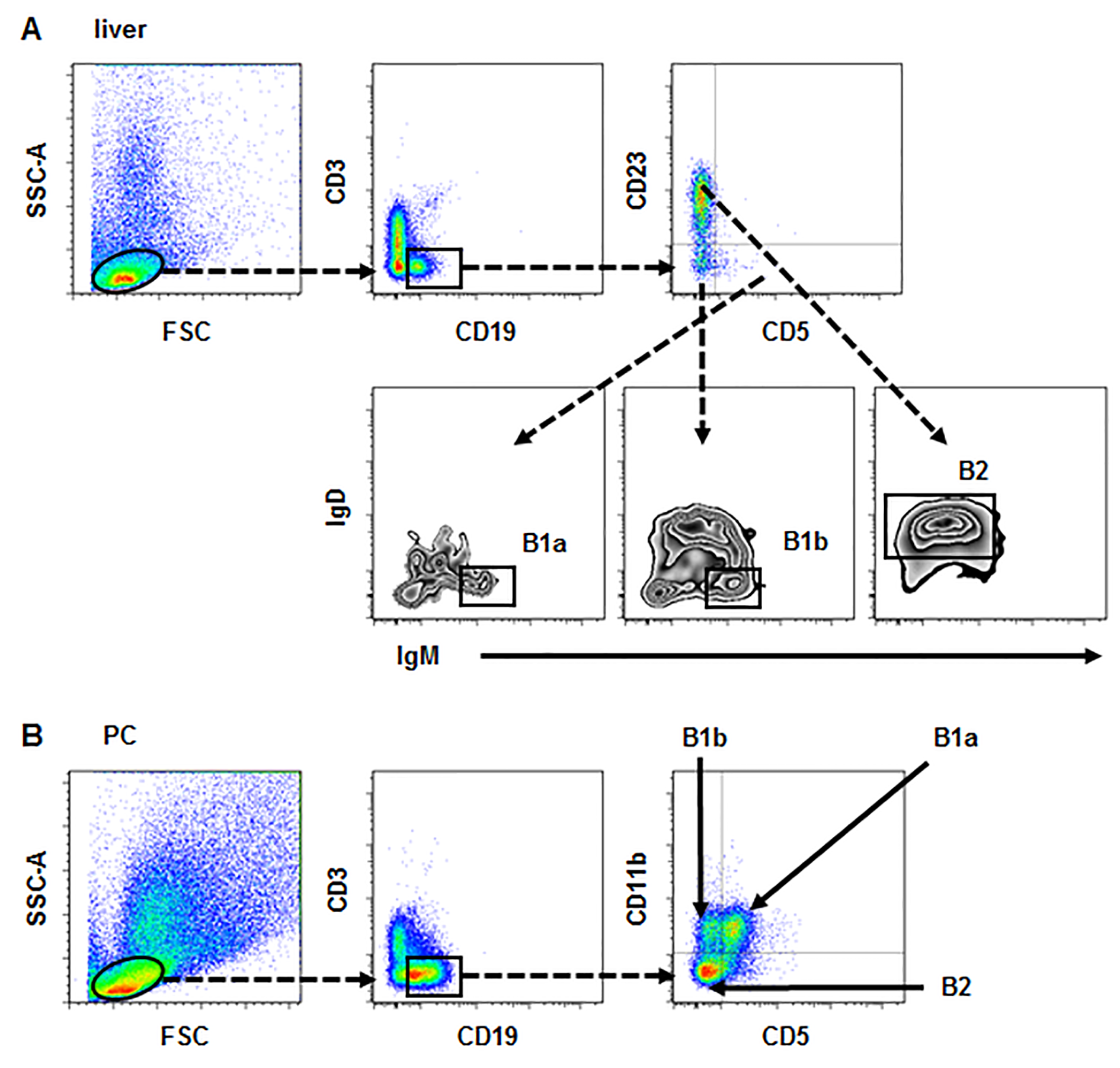

Supplement: S5 Fig — (A) Representative flow cytometry plots show the gating strategy to identify hepatic B1a cells (CD3−CD19+CD5+CD23−IgMhiIgDlo), B1b cells (CD3−CD19+CD5−CD23−IgMhiIgDlo), and B2 cells (CD3−CD19+CD5−CD23+IgMloIgDhi). (B) PC B1a cells were identified as CD3−CD19+CD5+CD11b+. PC B1b cells were identified as CD3−CD19+CD5−CD11b+. PC B2 cells were identified as CD3−CD19+CD5−CD11b−. (TIF) [file pntd.0007474.s006.tif]

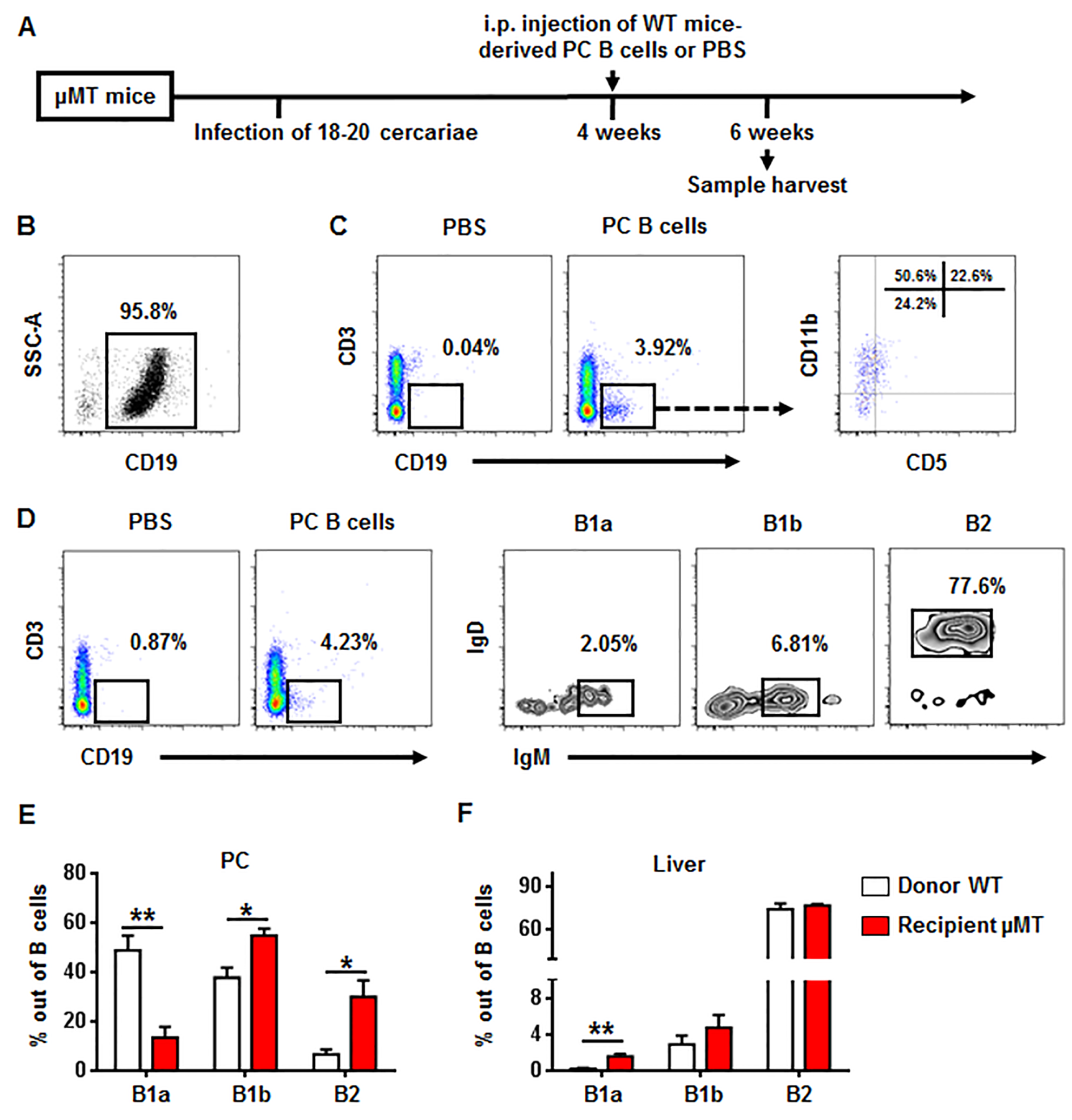

Supplement: S6 Fig — (A) μMT mice were infected with 18–20 cercariae of S. japonicum. MACS-sorted PC B cells (2 × 10^6^ cells) were purified from uninfected WT mice, and adoptive transferred into μMT mice 4 weeks after infection. Mice were sacrificed 6 weeks after infection. (B) Purity of PC B cells from WT mice after sorting. (C, D) Flow cytometric analysis of PC (C) and liver (D) B cell subsets after transfer in μMT mice. (E, F) The frequencies of B1a, B1b, and B2 cells in PC (E) and liver (F) of donor WT mice and recipient μMT mice. Data represent mean ± SD; n = 8–10 per group from two independent experiments. *p < 0.05, **p < 0.01. (TIF) [file pntd.0007474.s007.tif]
